# Development of Robust Lane-Keeping Algorithm Using Snow Tire Track Recognition in Snowfall Situations

**DOI:** 10.3390/s24237802

**Published:** 2024-12-05

**Authors:** Donghyun Kim, Yonghwan Jeong

**Affiliations:** 1 Department of Automotive Engineering, Seoul National University of Science and Technology, Seoul 01811, Republic of Korea; pdw03137@seoultech.ac.kr; 2 Department of Mechanical and Automotive Engineering, Seoul National University of Science and Technology, Seoul 01811, Republic of Korea

**Keywords:** autonomous driving, snowy roads, lane keeping, tire track detection, deep learning

## Abstract

This study proposed a robust lane-keeping algorithm designed for snowy road conditions, utilizing a snow tire track detection model based on machine learning. The proposed algorithm is structured into two primary modules: a snow tire track detector and a lane center estimator. The snow tire track detector utilizes YOLOv5, trained on custom datasets generated from public videos captured on snowy roads. Video frames are annotated with the Computer Vision Annotation Tool (CVAT) to identify pixels containing snow tire tracks. To mitigate overfitting, the detector is trained on a combined dataset that incorporates both snow tire track images and road scenes from the Udacity dataset. The lane center estimator uses the detected tire tracks to estimate a reference line for lane keeping. Detected tracks are binarized and transformed into a bird’s-eye view image. Then, skeletonization and Hough transformation techniques are applied to extract tire track lines from the classified pixels. Finally, the Kalman filter estimates the lane center based on tire track lines. Evaluations conducted on unseen images demonstrate that the proposed algorithm provides a reliable lane reference, even under heavy snowfall conditions.

## 1. Introduction

The Advanced Driving Assist System (ADAS) represents an advanced suite of technologies developed to recognize, evaluate, and respond to driving situations by controlling the vehicle. The primary goals of the ADAS include mitigating driver error, preventing collisions, and alleviating traffic congestion. Prominent examples of ADAS technologies include Autonomous Emergency Braking (AEB), Adaptive Cruise Control (ACC), Active Blind Spot Detection (ABSD), the Lane-Keeping Assist System (LKAS), and the Around View Monitor (AVM) [1,2,3,4]. The ADAS, which is designed to support drivers, ultimately aims to facilitate a transition toward fully autonomous driving. Recent advancements in the ADAS have not only improved driving convenience but also contributed to reductions in economic costs and pollution [5]. Despite these advantages, the transition to fully autonomous vehicles remains challenging. The complexity of traffic scenarios and the diversity of road conditions make the transition to fully autonomous driving difficult.

For vehicles without the ADAS, the driver must maintain constant vigilance over lane position and surrounding traffic, making long driving increasingly difficult. Driver fatigue reduces the concentration on lane keeping, increasing the risk of accidents. The LKAS addresses this issue by detecting lane departures and adding steering assistance to keep the lane. However, accurate and reliable lane detection remains challenging due to the diversity of road conditions [6]. In addition, complex lane markings and narrow lane widths further complicate lane detection problems [7].

Lane detection is crucial for identifying lanes on the road for intelligent vehicles. Detected lanes enable simpler functions, such as lane display for the driver, and more advanced tasks, like predicting lane changes to avert potential collisions. Cameras and LiDAR serve as foundational components in lane detection [8]. However. Even if these sensors are used, challenges persist when lane markings are obscured by factors such as other vehicles, wear, shadows, or inconsistent markings. Ongoing research is focused on developing robust lane detection and tracking algorithms under unfavorable conditions [9].

Lane detection methodologies are generally categorized into feature-based and model-based approaches. Feature-based algorithms utilize low-level features, such as lane edges. Known for their simplicity, robustness, and efficiency, these algorithms are well suited for real-time processing in complex environments without requiring prior information [10,11]. However, their sensitivity to weak lane markings or environmental noise can sometimes lead to suboptimal results. In contrast, model-based algorithms interpret lane geometry as curves, which helps mitigate the impact of weak markings and environmental noise. However, a model-based approach may lack adaptability across diverse driving environments [12,13,14]. Additionally, model-based algorithms generally demand higher computational resources compared to feature-based approaches.

To address lane detection challenges, systematic approaches have been introduced. A typical pipeline includes image preprocessing for noise reduction, feature extraction, and lane model fitting [15]. The primary aim of preprocessing is to minimize noise in lane images captured by onboard cameras. Techniques like RGB-to-HSV color conversion and Gaussian blurring help reduce image noise, providing a cleaner basis for feature extraction. During this stage, researchers apply various methods—including filtering techniques, Inverse Perspective Mapping (IPM), edge detection, and color space transformations—to isolate distinct lane features [16,17,18,19,20]. These techniques not only improve noise reduction but also enhance the precision of feature extraction. The extracted features are then fitted to mathematical models using methods like polynomial fitting, Hough transform, quadratic curve, hyperbolic, and spline approaches [21,22,23,24]. For tracking, techniques such as parabola equations, lane classification, and Kalman filters are employed to maintain lane continuity over time [25,26,27].

Recent developments in lane detection leverage Artificial Intelligence (AI) to simplify and automate the traditionally intensive processes of preprocessing, feature extraction, and model fitting. AI-based approaches primarily utilize Machine Learning (ML) and Deep Learning (DL) frameworks. ML provides computational efficiency through simpler models that work well with smaller datasets. However, ML often depends heavily on predefined features, which can require careful engineering and limit its adaptability to arbitrary patterns. Common ML methods include decision trees, support vector machines, and random forests [28,29,30].

Meanwhile, DL employs neural networks to achieve better accuracy where sufficient computational resources and data are available. Additionally, the complexity of DL models poses interpretability challenges, as they often function as “black boxes” [31,32,33]. Prominent DL architectures for lane detection include G-Net, ResNet50, and VGGNet16, each offering specific strengths and limitations suited to different pipeline stages [34,35,36,37]. For 3D lane detection, the concept of a virtual camera was introduced to address the limitations of monocular cameras [38]. This approach was developed in bird’s-eye view (BEV) conditions to align with the requirements of autonomous driving. Additionally, self-supervised learning was employed to ensure robust lane detection under diverse driving conditions [39].

Despite advancements in lane detection algorithms, the LKAS usually shows strong performance mainly on highways with clearer lane markings. However, LKAS performance deteriorates in challenging conditions, such as noisy or blurry lane markings and bad weather, including rain, snow, or fog. Several studies are investigating ways to improve lane detection in bad conditions. These include methods such as lane detection through color space conversion [40], enhanced segmentation of road boundaries for classification [41], vehicle trajectory prediction on snowy or icy surfaces [42], and navigation assistance on snow-covered roads using available road area data [43]. In addition, edge detection of lane markers in rainy or snowy conditions has been proposed using the comprehensive intensity threshold range [44]. Similarly, a method for detecting yellow lane markers by color analysis has been proposed in situations where they are covered with thin snow. However, only a few studies have been conducted on conditions where there is a lot of snowfall and no lane is visible at all. In particular, rather than including functions such as lane or object recognition through existing forward vision image processing, custom datasets are constructed by researchers themselves, and tire track recognition is implemented based on the custom datasets [40,43]. In response to these challenges, this study introduces a learning-based model that replaces conventional lane markings with tire tracks left by preceding vehicles on snow-covered roads, proposing a robust lane-keeping algorithm designed to function effectively in snowy conditions.

The contributions of this paper can be summarized as follows:

1. Multifunctionality in a Single Model: The proposed model integrates snow tire track detection while maintaining all the object detection functionalities of the camera system designed for autonomous driving.
2. Utilization of Publicly Available Driving Images: Instead of relying on self-acquired snow road data, the model leverages a variety of driving images sourced from publicly available datasets on the web. This approach enhances the model’s generalization ability to perform effectively under diverse driving conditions.
3. Lane Center Tracking via Filtering: After detecting tire tracks, a filtering-based tracking mechanism is implemented to estimate the lane center. This enables the vehicle to follow the lane center even when tire tracks exhibit high randomness.

## 2. Outline of Overall Process

**Figure 1:**
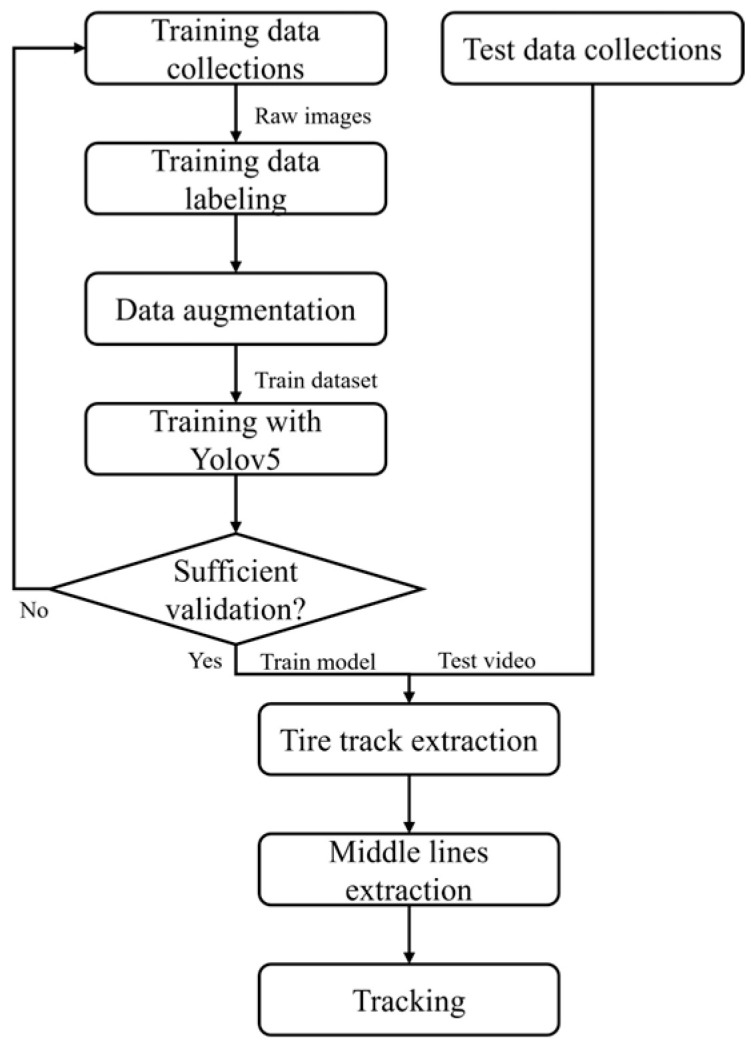
Workflow of the proposed algorithm.

The development of a YOLOv5-based snow tire track detection model and a robust lane-keeping algorithm utilizing a Kalman filter follows the workflow illustrated in Figure 1. Initially, video data is collected and annotated to construct a comprehensive dataset for training the YOLOv5 model for tire track detection on snowy roads. During the training phase, performance metrics, including *Precision* and *Recall*, are continuously monitored to ensure the model achieves accuracy. After training, the model undergoes evaluation on a set of test images to assess its effectiveness in detecting tire tracks in snowy conditions. Detected tire track images are then preprocessed to enhance visual quality, followed by feature extraction and fitting with a quadratic curve to accurately represent the lane structure. Finally, the centerline of the fitted curve is estimated and tracked over time using a Kalman filter.

## 3. Tire Track Detection Model

### 3.1. Data Collections

DL requires three datasets—training, validation, and testing—for effective model development. The training dataset is crucial for learning patterns between input and output features, the validation dataset assesses model performance during training to avoid overfitting or underfitting, and the testing dataset evaluates the model on unseen data. This chapter discusses the composition and acquisition methods for these datasets in developing the lane-keeping algorithm. A robust training dataset must be diverse, representative, sufficiently large, high-quality, and balanced. These characteristics ensure the model generalizes well to real-world scenarios.

**Figure 2:**
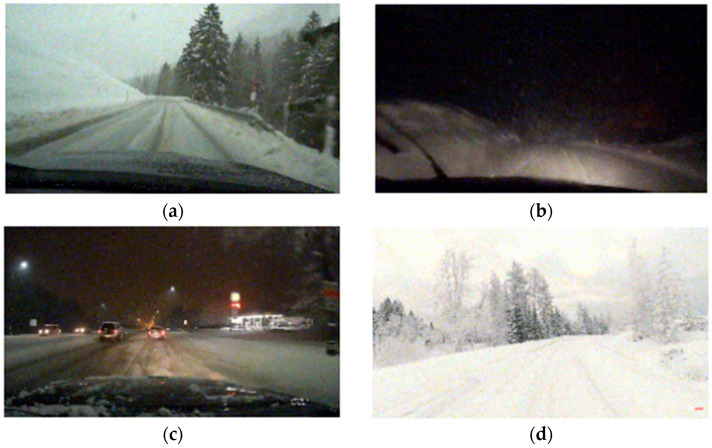
Examples of the collected datasets. (**a**) Nominal case; (**b**) snowy and night case; (**c**) preceding vehicle case; (**d**) blurry case.

The appearance of tire tracks on snow varies due to factors such as lighting, road type, snowfall, and traffic. Lighting affects track brightness, preceding vehicles influence track length, and snow depth determines track depth. Road types—like mountain roads, city streets, or highways—further shape the track patterns, which are also altered by snowfall and repeated traffic. Collecting accurate data under such conditions is challenging. To streamline this process, video footage was sourced online, providing a wide variety of scenarios. As shown in Figure 2, (a) clear tire tracks in snowy daylight provide baseline data; (b) nighttime conditions capture blurred, color-variable tracks; (c) shorter tracks with mild curvature appear in urban settings; and (d) brighter conditions reveal variations in depth, color, and brightness.

More detailed information on video sources and content is as follows. The custom datasets are derived from three distinct video sources available online. The first video on YouTube captures a scene of snow conditions on two-lane mountain roads [45]. It features varying depths and quantities of tire tracks caused by significant traffic. The second video, available on YouTube, depicts nighttime mountain roads with heavy snow, where low traffic results in faint tire tracks amid ongoing snowfall [46]. The third video, also on YouTube, showcases a snowy daytime scene in Switzerland, with visible road surfaces under light snowfall [47]. These videos, recorded in diverse spatial and situational contexts, contribute to a snow dataset that reflects a wide range of environmental conditions, ensuring high variability and comprehensiveness.

**Figure 3:**
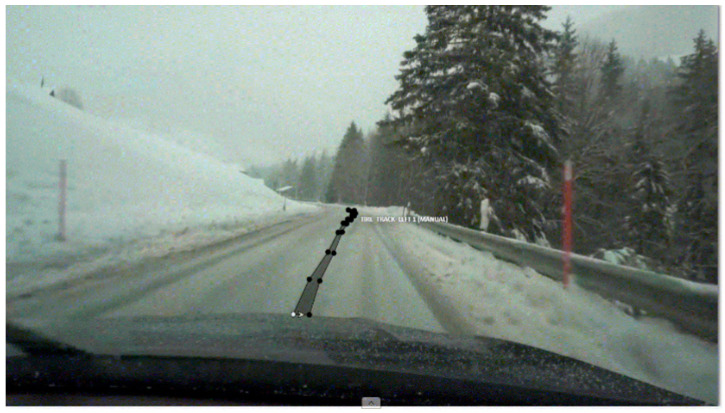
Example of the data labeling using CVAT.

The video data were segmented into individual frames and annotated using the Computer Vision Annotation Tool (CVAT). The CVAT was chosen for its intuitive interface, server-based access, and tracking mode, which significantly reduced labeling time and enhanced accuracy. For instance, a 10 min video generated approximately 3600 frames. Given the slow driving speeds in snowy conditions, this equated to data covering short distances. CVAT’s tracking mode minimized frame-by-frame labeling by automatically labeling objects across adjacent frames, improving efficiency. Figure 3 shows the labeling example using CVAT. 

Despite these advantages, the labeled dataset volume remained insufficient. To address this, data augmentation was applied to expand and diversify the dataset. Augmentation techniques included flipping, zooming, brightness adjustment, blur addition, noise, and rotation. These methods replicated real-world conditions, improving the model’s robustness and adaptability. Table 1 details the specific augmentation techniques used. Blur was applied in units of 1 pixel, and noise was applied with a 3% reduction error of the original color value. And whether flip, blur, and noise were applied or not was randomly extracted with a 50% probability. In the case of zoom, brightness, and rotation, it was applied by random extraction using uniform distribution within the range presented in the table. In this way, the total tire track label data were tripled by performing the random application of the six data augmentation methods twice per image.

The dataset obtained through data augmentation includes labels for both tire tracks and vehicles. However, the data including vehicles are underrepresented, necessitating an increase in vehicle data samples. To avoid extensive manual labeling for balancing tire track and car label ratios, the Udacity dataset was integrated to supplement the vehicle data. The Udacity dataset contains 97,942 labels across 11 classes and 15,000 images. From this set, 7152 images were randomly selected and merged with the tire track dataset. During this process, a unique class number was assigned for tire tracks, while the original label from the Udacity dataset was retained. This approach yielded a comprehensive dataset of 12,100 images, consisting of 7152 images from the Udacity dataset and 4948 tire track images. Datasets are divided into training, validation, and test sets in an 8:1:1 ratio.

### 3.2. Training with YOLOv5

This study employs YOLOv5 for tire track detection. YOLO (You Only Look Once) is a prominent object detection algorithm that predicts bounding boxes and classes for identified objects. The YOLO model has evolved through multiple versions, from 1 to 8, each update bringing significant performance improvements. Versions 1 to 4, built on the Darknet framework, pose setup challenges due to their specific development environment. Meanwhile, versions 5 to 8—implemented in PyTorch 1.7—use an enhanced Convolutional Neural Network (CNN) and simplified development process. These PyTorch-based versions have notably reduced computation times. Memory usage is also optimized through compressed pooling and feature pyramid operations. Among the various versions of YOLO, YOLOv5 was selected for this study.

YOLOv5 offers four algorithmic versions—YOLOv5s, YOLOv5m, YOLOv5l, and YOLOv5x—each with distinct performance attributes, as outlined in [48]. YOLOv5m was chosen for this study based on a comparison of GPU performance, validation accuracy, and GPU latency in relation to the available hardware. Since the goal of this study is to detect snow tire tracks while maintaining the object detection function provided by YOLO, the default architecture was used without change. The details of the network structure of YOLOv5m were given in [48]. Therefore, for snow tire track detection, only the batch size and the number of epochs for learning need to be determined.

Choosing suitable batch sizes and epochs is essential for evaluating a model’s performance and confirming its suitability for the given task. Model performance is typically assessed using metrics such as accuracy, *Precision*, *Recall*, *F1 score*, and mean Average Precision (*mAP*). In this study, the optimal batch size and number of epochs for training the custom dataset were determined based on *Precision*, *Recall*, *F1 score*, and *mAP*. The metrics for model evaluation were defined as follows:

(1) $$\begin{array}{l} Precision=\frac{TP}{TP+FP}\\ Recall=\frac{TP}{TP+FN}\\ F1\;score=2\cdot \frac{Precision\cdot Recall}{Precision+Recall}\\ mAP=\frac{1}{k}\sum_{i=1}^{k}AP_{i}\\ where,\;\;TP=\mathrm{True}\;\;\mathrm{Positive}\\ \;\;\;\;\;\;\;\;\;FP=\mathrm{False}\;\;\mathrm{Positive}\\ \;\;\;\;\;\;\;\;\;FN=\mathrm{False}\;\;\mathrm{Negative} \end{array}$$

**Figure 4:**
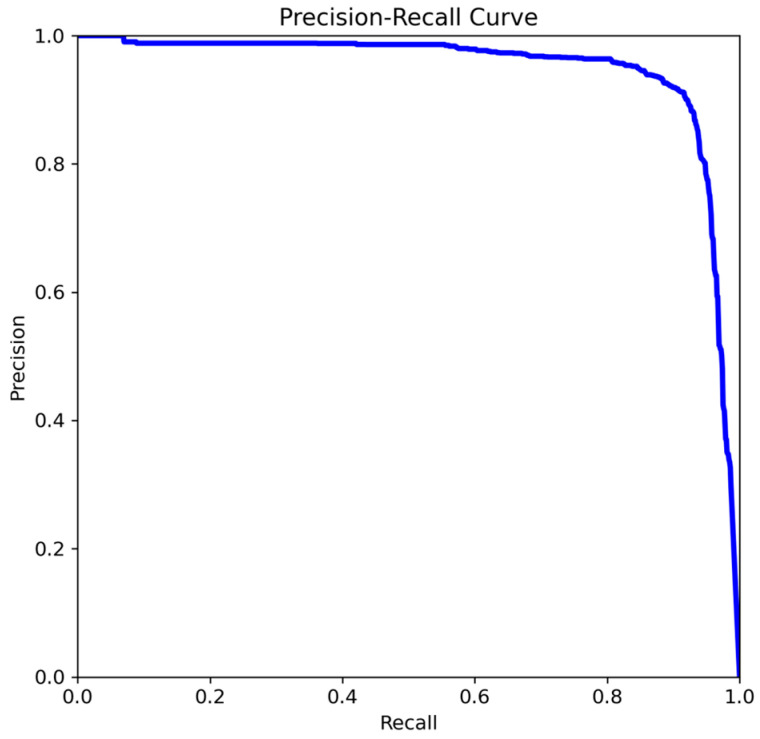
PR curve of trained model.

In addition to the metrics, the precision–recall (PR) curve is used to evaluate the model. While the PR curve is effective for understanding an algorithm’s overall performance, it is less suitable for quantitatively comparing different algorithms. To facilitate this, *mAP* is used as a performance indicator. The *F1 score* provides a balanced evaluation by considering both metrics equally, avoiding favor toward either *Precision* or *Recall*. Due to potential data imbalance in the custom-configured training dataset, the *F1 score* was also incorporated as an essential evaluation metric. Following a comparative analysis of the model’s performance across varying batch sizes and epochs, the optimal configuration was identified as a batch size of 16 and 100 epochs. Figure 4 presents the PR curve for the model trained with a batch size of 16 and 100 epochs. Table 2 summarizes the performance metrics of the proposed tire track detection model.

## 4. Lane Center Estimator

### 4.1. Tire Track Extractions

The process for extracting the tire track can be summarized into three steps. First, the learned YOLOv5 model is applied to extract pixels with tire tracks. After that, skeletonization was performed to extract the center point of the tire track with respect to the vehicle’s traveling direction. Finally, the center points were clustered and fitted with a quadratic curve. A detailed description of each step is as follows.

**Figure 5:**
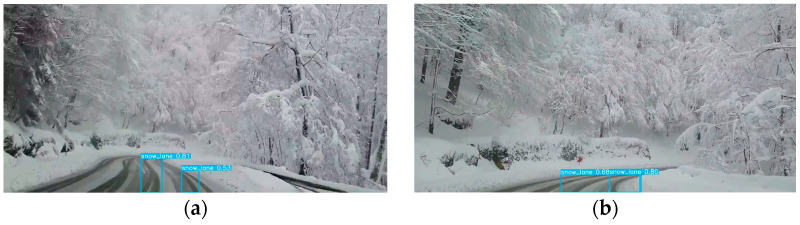

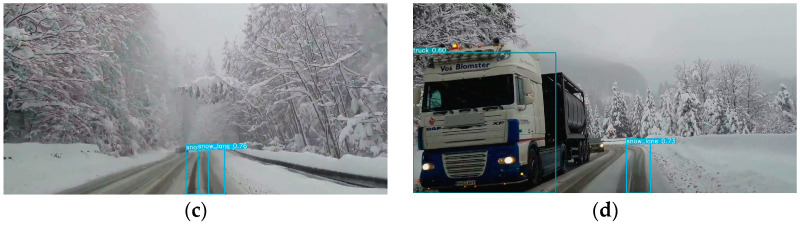
Results of applying the proposed model to the test image. (**a**) Case 1; (**b**) Case 2; (**c**) Case 3; (**d**) Case 4.

The YOLOv5-based learning model was applied to test images sourced from an online video platform. Figure 5 displays the results of YOLOv5 applied to these test images, where tire tracks were clearly identifiable. While YOLOv5 includes a cropping function, using it for tire track extraction poses challenges: it complicates the identification of track origins and tends to capture all visible tire tracks, including those not relevant to lane keeping, thereby introducing extraneous data. To mitigate this, only tire tracks essential for lane keeping were selected based on their assigned labels, ensuring a focused extraction from the full image.

**Figure 6:**
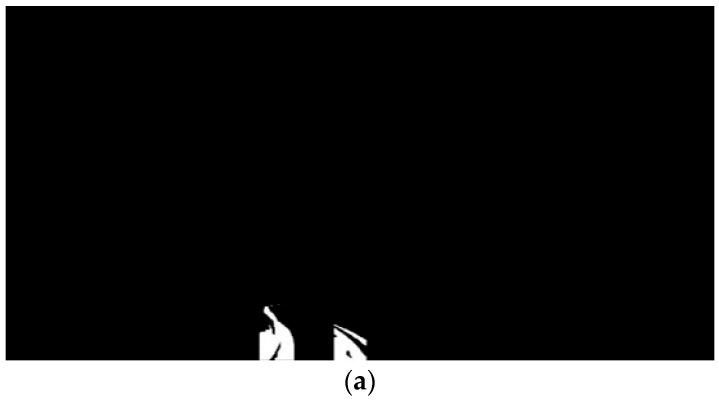

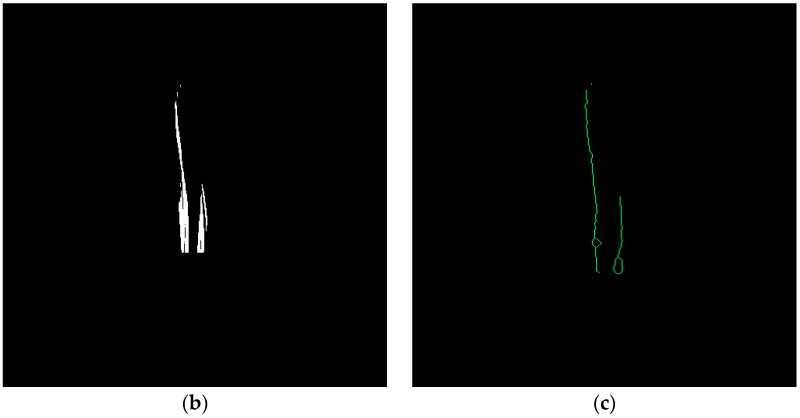
Middle line detection in binarized BEV. (**a**) Cropped tire track; (**b**) transformation to BEV; (**c**) detected middle line.

For computational efficiency, tire tracks were classified into brightness and saturation levels and then converted to grayscale. As shown in Figure 6a, a Gaussian blur was applied to the grayscale tire track image, followed by binarization to reduce image noise. The binarized image then underwent IPM, a technique that removes perspective distortion to produce a BEV of the scene. This conversion was achieved using the mathematical framework of (2).

(2) $$s\left[\begin{array}{c} u\\ v\\ 1 \end{array}\right]=\left[\begin{array}{ccc} f_{x} & 0 & u_{0}\\ 0 & f_{y} & v_{0}\\ 0 & 0 & 1 \end{array}\right]\left[\begin{array}{cccc} r_{11} & r_{12} & r_{13} & t_{1}\\ r_{21} & r_{22} & r_{23} & t_{2}\\ r_{31} & r_{32} & r_{33} & t_{3} \end{array}\right]\left[\begin{array}{c} X\\ Y\\ Z\\ 1 \end{array}\right]$$

The transformation equation aligns the ground coordinate system with the vehicle’s motion and camera coordinates. Each pixel in the image provides input data, which are first processed through a matrix that converts from the camera’s coordinate system to the vehicle’s coordinate system. This result is then multiplied by a matrix to account for the vehicle’s roll, yaw, and pitch, ultimately aligning the image with the ground coordinate system. An example of the IPM-converted image is depicted in Figure 6b.

For lane keeping, the middle line of the tire tracks is identified from the BEV image. Tire tracks, being wider and more varied than standard lane markings, make it necessary to isolate only the relevant tracks. Figure 6c illustrates the extraction of these middle lines using the Skeletonize3D technique, which accurately derives the middle line from irregularly shaped tire tracks. After extracting this middle line, the image is cropped to 100 × 500 pixels and rotated by 90° to facilitate polyfitting for tire track representation. Extracting the middle line prior to cropping avoids quality degradation and noise amplification.

**Figure 7:**
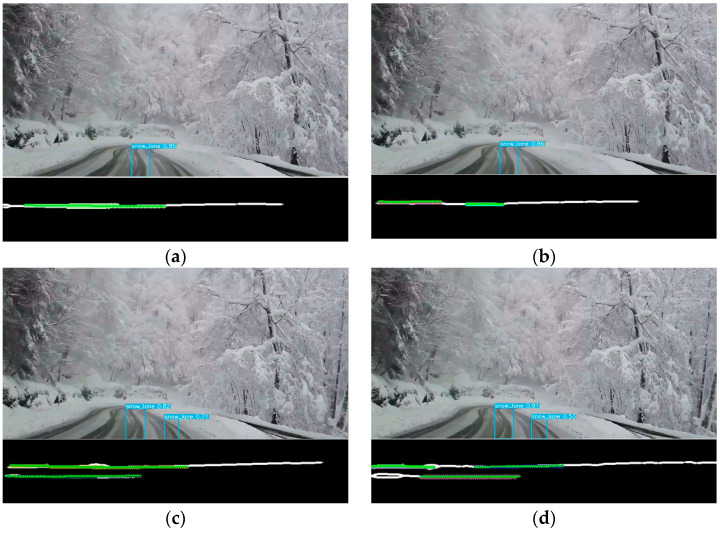

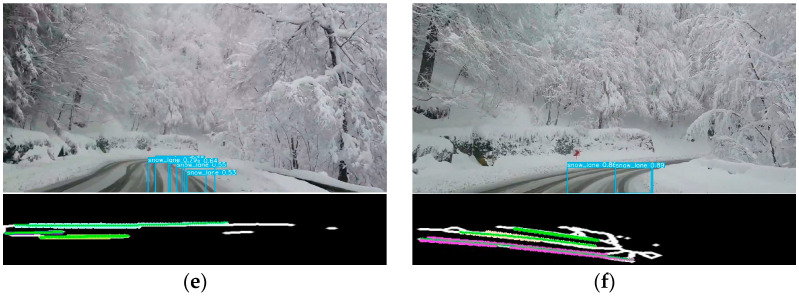
Detection results of the middle line of tire track cluster using DBSCAN. (**a**) Single-cluster case #1; (**b**) single-cluster case #2; (**c**) two-cluster case #1; (**d**) two-cluster case #2; (**e**) multiple-cluster case; (**f**) adjacent-lane cluster case.

Using the cropped image, representative lines are identified from the extracted middle line using the Hough transform. The transform simplifies tire track lines into points, subsequently clustered through the DBSCAN algorithm. A quadratic polynomial function is fitted to each cluster, identifying the tire tracks relevant to the lane-keeping algorithm. Due to the IPM’s reliability in short distances near the camera and unreliability at greater distances, a second-order polynomial fitting is preferred over higher orders. Figure 7 shows the polyfitted line in green, with clustered points represented in magenta.

### 4.2. Lane Center Estimation Using Kalman Filter

To make the vehicle follow the center of the lane, extracting the lane center from the identified tire track’s middle lines is important. If the snow tire tracks exist as lane markers, the mean of the quadratic curve representing the tire track’s midline is the lane center. However, due to the varying number and locations of clusters extracted via DBSCAN, it is difficult to determine the center of the lane as an average for the left and right tire tracks. Therefore, it is necessary to establish the selection criteria for highly tire track midline clusters first.

**Figure 8:**
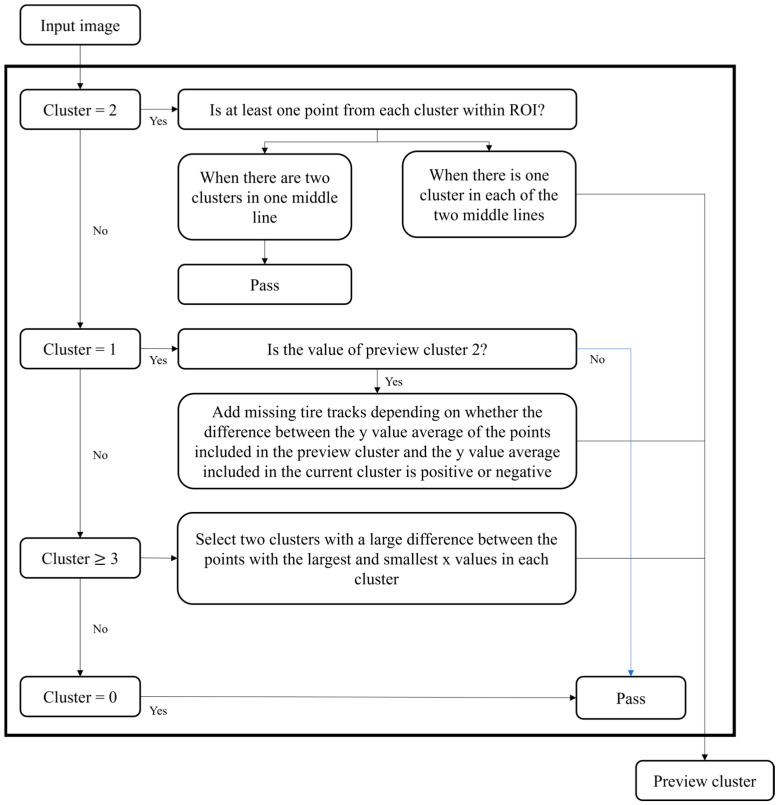
Decision tree for cluster selection.

Before describing the cluster selection criteria, the new concept, preview cluster, is introduced to ensure the continuous monitoring of the left and right tire tracks. The selected clusters for lane center estimation are named as preview cluster. The preview cluster is initialized and updated based on the count and characteristics of clusters representing tire tracks, as depicted in Figure 8. In scenarios where two clusters were recorded in the previous step but only one is detected in the current step, the missing tire track can be inferred and reconstructed using data from the previous step by shifting the detected tire track laterally by the interval between the two tire tracks of the previous step. This update mechanism ensures the accurate lane center estimation across varying detection conditions.

Figure 8 illustrates a decision tree designed to systematically select the suitable tire track cluster based on varying road conditions. A 100 × 100-pixel region near the camera in the detected image is designated as the Region of Interest (ROI). Depending on the number of clusters identified within the ROI, distinct scenarios emerge. As described in Figure 8, the case where the number of clusters is two is considered first. When two clusters are present, there are two possible configurations: the first has one cluster each on the left and right tire tracks, both clusters are assigned as preview clusters that calculate the lane center line. Meanwhile, if two clusters are extracted from a single tire track, the preview cluster is not updated. Next, if the number of the cluster within the ROI is one, the opposite cluster is created virtually. If the preview cluster has not been determined in the previous step, the preview cluster is not updated.

Then, if the number of the cluster is three or more, the two clusters showing the largest x-coordinate difference among points in each cluster are selected for preview cluster updating. In this case, two additional conditions are considered. Then, two situations can arise: one where unwanted cluster result from overlapping lanes, as shown in Figure 7e, and another where adjacent lanes appear in the BEV image, as shown in Figure 7f. First, overlapping tire tracks within a cluster tend to be closer, it is easily distinguished by y-coordinate differences. Second, if a tire track from an adjacent lane is detected, it can be filtered out by the x-coordinate difference, as adjacent lane tracks typically appear shorter in a BEV due to the Field of View (FOV) of the camera. Lastly, no clusters are found in the ROI, which indicates that no recognizable tire marks are present, signifying a lack of usable data for lane keeping.

**Figure 9:**
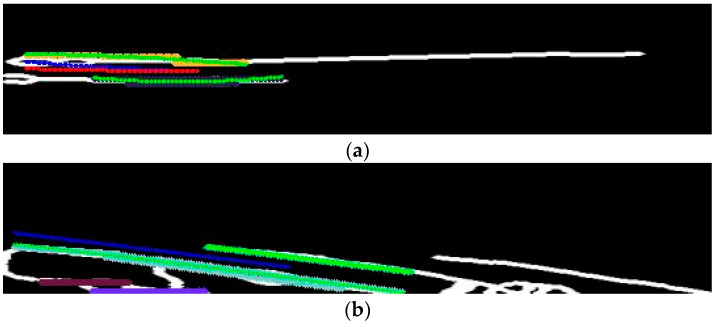
Bias occurrence cases. (**a**) Bias to the longer tire track; (**b**) bias due to the adjacent lane tire track.

After selecting the two clusters, which are called the preview cluster, the Kalman filter is applied to continuously estimate and track the lane center. Unlike lane markers, tire tracks exist irregularly, so bias occurs when the average of the currently detected cluster is simply taken. As shown in Figure 9a, simple averaging resulted in a bias toward longer tire tracks, as shown by the blue line. Additionally, if three or more tire tracks were extracted due to the tire track from the adjacent lane, as shown in Figure 9b, the lane center could be biased to the adjacent lane tire track, as shown by the blue line. To address this issue, a Kalman filter was applied to compute a new lane center, depicted as the red line. The Kalman filter not only mitigates these biases but also allows for continuous lane center tracking. By smoothing the lane center position, it reduces the influence of longer tire track biases and prevents the misidentification of adjacent lane tracks, contributing to more stable and accurate lane keeping. Since the tire track is modeled as a quadratic curve, the process and measurement model of the Kalman filter is defined as follows:

(3) $$\begin{array}{ll} \dot{x} & =Ax+Bu+w\\ & =\left[\begin{array}{ccc} 0 & 0 & 0\\ 2v & 0 & 0\\ 0 & v & 0 \end{array}\right]\left[\begin{array}{c} a_{2}\\ a_{1}\\ a_{0} \end{array}\right]+\left[\begin{array}{c} 0\\ -1\\ 0 \end{array}\right]\gamma +w\\ w & \;\sim \;N\left(\begin{array}{cc} 0 & Q \end{array}\right) \end{array}$$

(4) $$\begin{array}{ll} z & =Hx+v\\ & =\left[\begin{array}{ccc} 1 & 0 & 0\\ 0 & 1 & 0\\ 0 & 0 & 1 \end{array}\right]x+v\\ v & \sim N\left(\begin{array}{cc} 0 & V \end{array}\right) \end{array}$$

where *v* and γ are the longitudinal velocity and yaw rate of the vehicle. **w** and **v** are the process and measurement noise.

## 5. Results

**Figure 10:**
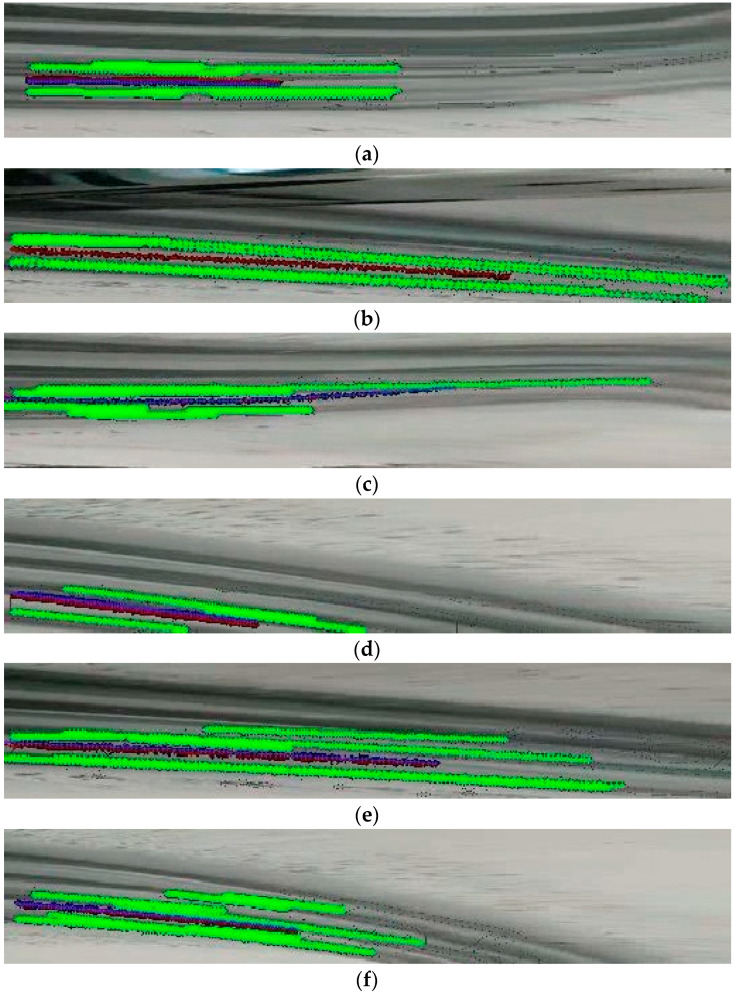
Tire track detection and lane center estimation results. (**a**) Case A; (**b**) case B; (**c**) case C; (**d**) case D; (**e**) case E; (**f**) case F.

The evaluation results of the proposed algorithm are summarized in Figure 10, which depicts the scenario of a vehicle traveling from left to right. In Figure 10, the recognized tire track area, as captured by the front camera and converted to a BEV, is highlighted in green, while the lane center line estimated from the tire tracks is marked in blue. As shown in Figure 10a, the proposed algorithm effectively detects the tire tracks on both sides of the vehicle. Since the tire tracks become indistinct beyond the midpoint of the longitudinal direction, the estimated lane center is generated only within the detected tire track length.

Case B mirrors case A but features tire tracks detected over a greater distance, resulting in a longer estimated center line compared to case A. Figure 10c illustrates case C, where an asymmetric tire track scenario is presented. Here, the tire track appears thick near the vehicle and gradually thins with distance, with the left-side track extending further than the right. Despite this asymmetry, the algorithm correctly estimates the required lane center for lane maintenance.

Case D, depicted in Figure 10d, involves a sharp curvature in the road where only partial tire track visibility is achieved within the front camera’s sensing area. Even in this challenging scenario, the algorithm detects most of the asymmetric left and right tire tracks within the camera’s converted BEV, and the center lane is accurately estimated using the partial data. Lastly, cases E and F depict scenarios where parts of the opposite lane’s tire tracks are detected. As the opposite lane enters portions of the FOV, only specific segments of the opposite lane’s tire tracks are recognized. However, through the application of the center lane extraction filter rule, these tracks are excluded, ensuring that the center lane line is correctly estimated.

The analysis results for the six cases are summarized as follows. First of all, looking at the performance according to the level of the curve of the road, roads close to straight lines can be seen, such as cases A, B, C, and E, as well as situations where the heading and curvature of the road are large, such as case D and F. In addition, it can be seen that the lane to follow, not the opposite lane, was estimated through lane tracking using the Kalman filter, even in cases E and F, where the location of the vehicle deviated from the current driving lane. Figure 10 shows the central estimation performance of a robust car even when the clarity and width of the tire track change. In particular, it can be used in various models since not only the snow driving data obtained from the web but also open datasets such as Udacity were used together for the composition and verification of the dataset.

## 6. Conclusions

In this study, we developed an algorithm to leverage tire tracks on snow-covered roads for effective lane keeping. The approach involved constructing a comprehensive learning dataset with diverse images of visible tire tracks, which were augmented with open-source datasets to simulate various real-world conditions. Using YOLOv5, the algorithm was trained and evaluated based on key performance metrics, including the PR curve, *mAP, Precision, Recall*, and *F1 score*. The evaluation results demonstrated the algorithm’s robustness and effectiveness in detecting and utilizing tire tracks for lane keeping under challenging snow conditions.

To address issues such as lane marker misidentification and non-detection, corrective measures were implemented, including the integration of a Kalman filter to estimate the lane center and enhance tracking reliability. This research makes a significant contribution by demonstrating the feasibility of using tire tracks as substitutes for traditional lane markings in snowy environments. By mitigating lane deviations caused by obscured markings due to snow accumulation, our approach has the potential to improve road safety and reduce accident rates in adverse weather conditions.

Future research will focus on integrating the lane-keeping algorithm with existing vehicle technologies, such as Lane-Keeping Assist Systems (LKAS) and Adaptive Cruise Control (ACC). This integration aims to enable vehicles to autonomously maintain lanes under snowy conditions, thereby reducing driver fatigue and enhancing safety across extensive road networks, particularly in regions prone to heavy snowfall.

## Figures and Tables

**Table 1:** Methods and parameters of data augmentation.

Method	Degree of Application	Sampling	Reason for Choosing
Flip	Horizontal	Binary Selection	Similar to real-life situations
Blur	1 px		Similar to an out of focus situations
Noise	3%		Similar to snowfall conditions
Zoom	0 to 50%	Uniform Distribution	Change in camera image settings and installation
Brightness	−40 to 40		Change in illuminance over time
Rotation	−3° to 3°		Change in camera installation

**Table 2:** Performance of the tire track detection model.

Measures	Values
Precision	0.907
Recall	0.735
F1-score	0.812
mAP	0.88

## Data Availability

Data are available upon request from the authors.
